# 8-Meth­oxy-3,3,5-trimethyl-3,11-dihydro­pyrano[3,2-*a*]carbazole

**DOI:** 10.1107/S160053681002074X

**Published:** 2010-06-05

**Authors:** C. Uvarani, P. Ramesh, K. Ravichandran, P. S. Mohan, M. N. Ponnuswamy

**Affiliations:** a Department of Chemistry, School of Chemical Sciences, Bharathiar University, Coimbatore 641 046, India; b Centre of Advanced Study in Crystallography and Biophysics, University of Madras, Guindy Campus, Chennai 600 025, India

## Abstract

In the title compound, C_19_H_19_NO_2_, commonly called koenimbine, the pyran ring adopts a sofa conformation. The carbazole ring system is planar [r.m.s. deviation = 0.063 (1) Å]. A *C*(10) zigzag chain running along the *b* axis is formed through inter­molecular C—H⋯O hydrogen bonds. The chains are linked *via* weak C—H⋯π and N—H⋯π inter­actions.

## Related literature

For bond-length data, see: Allen *et al.* (1987 ▶). For the bio­logical activity of carbazole derivatives, see: Kong *et al.* (1986 ▶); Ito (2000 ▶); Ramsewak *et al.* (1999 ▶); Chowdhury *et al.* (2001 ▶); Fiebi *et al.* (1985 ▶). For puckering parameters, see: Cremer & Pople (1975 ▶). For asymmetry parameters, see: Nardelli (1983 ▶). For hydrogen-bond motifs, see: Bernstein *et al.* (1995 ▶).
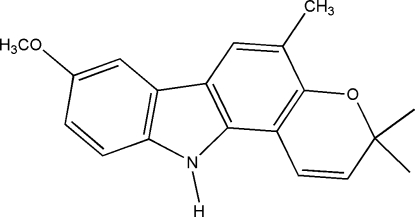


## Experimental

### 

#### Crystal data

C_19_H_19_NO_2_
*M* *_r_* = 293.35
Monoclinic, 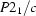

*a* = 8.290 (5) Å
*b* = 8.693 (5) Å
*c* = 21.326 (5) Å
β = 90.742 (5)°
*V* = 1536.7 (13) Å^3^
*Z* = 4
Mo *K*α radiation
μ = 0.08 mm^−1^
*T* = 293 K
0.20 × 0.17 × 0.16 mm

#### Data collection

Bruker SMART APEXII area-detector diffractometer
Absorption correction: multi-scan (*SADABS*; Bruker, 2008 ▶) *T*_min_ = 0.984, *T*_max_ = 0.987
14325 measured reflections
3803 independent reflections
3050 reflections with *I* > 2σ(*I*)
*R*_int_ = 0.026

#### Refinement

*R*[*F* ^2^ > 2σ(*F* ^2^)] = 0.046
*wR*(*F* ^2^) = 0.137
*S* = 1.05
3803 reflections
207 parameters
H atoms treated by a mixture of independent and constrained refinement
Δρ_max_ = 0.24 e Å^−3^
Δρ_min_ = −0.22 e Å^−3^

### 

Data collection: *APEX2* (Bruker, 2008 ▶); cell refinement: *SAINT* (Bruker, 2008 ▶); data reduction: *SAINT*; program(s) used to solve structure: *SHELXS97* (Sheldrick, 2008 ▶); program(s) used to refine structure: *SHELXL97* (Sheldrick, 2008 ▶); molecular graphics: *ORTEP-3* (Farrugia, 1997 ▶); software used to prepare material for publication: *SHELXL97* and *PLATON* (Spek, 2009 ▶).

## Supplementary Material

Crystal structure: contains datablocks global, I. DOI: 10.1107/S160053681002074X/sj5011sup1.cif

Structure factors: contains datablocks I. DOI: 10.1107/S160053681002074X/sj5011Isup2.hkl

Additional supplementary materials:  crystallographic information; 3D view; checkCIF report

## Figures and Tables

**Table 1:** Hydrogen-bond geometry (Å, °) *Cg*1 is the centroid of the N1/C2/C7/C8/C16 ring and *Cg*4 is the centroid of the C8–C11/C15/C16 ring.

*D*—H⋯*A*	*D*—H	H⋯*A*	*D*⋯*A*	*D*—H⋯*A*
C3—H3⋯O1^i^	0.93	2.54	3.333 (2)	143
N1—H1⋯*Cg*4^ii^	0.872 (18)	2.744 (17)	3.528 (2)	149.7 (14)
C17—H17*C*⋯*Cg*1^iii^	0.96	3.08	3.489 (3)	107
C17—H17*C*⋯*Cg*4^iii^	0.96	3.00	3.514 (3)	115

Symmetry codes: (i) 
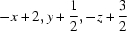
; (ii) 
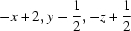
; (iii) 
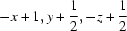
.

**Fig. 1.**
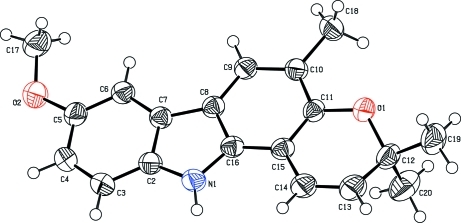
The molecular structure of the title compound, showing the atomic numbering and with displacement ellipsoids drawn at the 50% probability level.

**Fig. 2.**
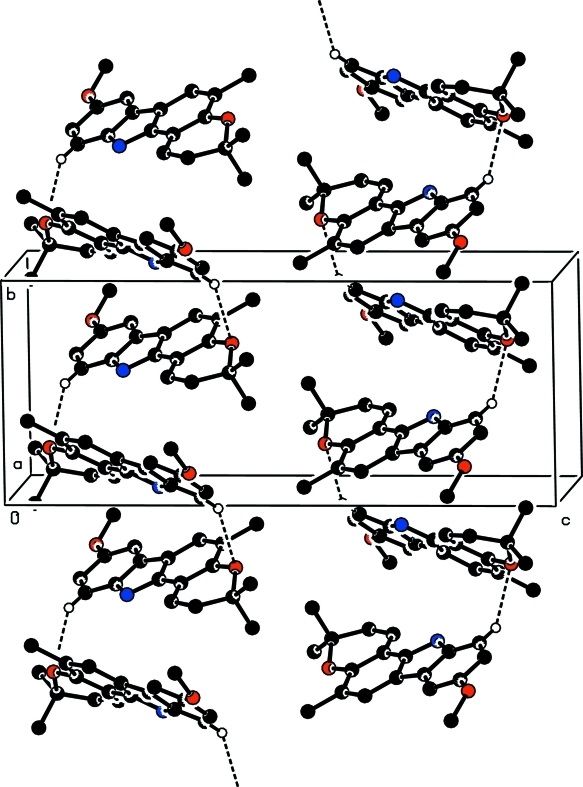
The crystal packing of the title compound. H atoms not involved in hydrogen bonding (dashed lines) have been omitted for clarity.

## supplementary crystallographic information

### Comment

*Murraya koenigii (L.)* Spreng (Family of Rutaceae), commonly known as the Indian curry leaf plant, is cultivated for the aromatic and appetizing nature of its leaves. The leaves are used for flavouring southern Asian dishes. Various parts of the plant have been used in traditional or folk medicine for the treatment of head-, tooth-, and stomach aches, influenza, rheumatism, traumatic injury, insect and snake bites, and also as an antidysentric as well as an astringent (Kong *et al.*, 1986). Recently, several biological activities have been reported for carbazole alkaloids. Bioactive coumarins, acridone alkaloids and carbazole alkaloids from the family of Rutaceae were reviewed (Ito, 2000). Mahanimbine, murrayanol, and mahanine compounds isolated from *M. koenigii* exhibit antioxidant, mosquitocidal and antimicrobial activities (Ramsewak *et al.*., 1999). Activities of carbazoles from *M. koenigii* against Gram-positive and Gram-negative bacteria and fungi were reported (Chowdhury *et al.*, 2001). The ethanol extract of *M. koenigii* displayed cytotoxic activity against cultured KB cells (Fiebi *et al.*, 1985). Against this background and to ascertain the structure and molecular conformation, the X-ray structure determination of the title compound has been carried out.

An ORTEP plot of the molecule is shown in Fig. 1. The carbazole ring system is planar (r.m.s. deviation 0.016 Å). The pyran ring in the molecule adopts sofa conformation with the puckering parameters (Cremer & Pople, 1975) and asymmetry parameters (Nardelli, 1983):  q_2_ = 0.287 (1) Å, q_3_ = -0.126 (1) Å, φ_2_ = 136.6 (3)° and Δ_s_(C12 & C15)= 10.9 (2)°. The N—C bond lengths, namely N1—C2 and N1—C16 [1.386 (2) & 1.383 (2) Å] deviate slightly from the mean value reported in the literature 1.370 (12) Å (Allen *et al.*, 1987). The sum of the bond angles around N1 [359.3°] is in accordance with *sp*^2^ hybridization. The methoxy group substituted at C5 deviates slightly from the plane of the attached carbazole ring system [C6—C5—O2—C17 = 19.3 (2)°].

The crystal packing of the molecules is controlled by C–H···O and C—H··· π types of intermolecular interactions. Atom C8 of the molecule at (x, y, z) donates a proton to atom O1 of the molecule at (-x+2, y+1/2, -z+1/2+1), which form a one dimensional zigzag C(10) chain (Bernstein *et al.*, 1995) running along the *b*–axis, Fig. 2. The packing of the molecules is further influenced by C—H··· π contacts and an N1—H1···Cg4 interaction of Table 1; Cg4 is the centroid of the C8/C9/C10/C11/C15/C16 benzene ring.

### Experimental

The air dried fruit pulps of *M. koenigii* were extracted with n-hexane in a Soxhlet apparatus. The total extract was concentrated and kept at room temperature. A dirty white solid separated out. This was dissolved in chloroform and chromatographed using a silica gel column and eluted successively with n-hexane and n-hexane- chloroform mixture. The fraction obtained with 7% chloroform in hexane afforded a white crystalline solid. Which on repeated crystallization with methanol:chloroform (3:1) as solvent afforded white crystalline solid koenimbine (3,11-Dihydro-8-methoxy-3,3,5- trimethylpyrano[3,2-a]carbazole).

### Refinement

The N-bound H atom was located in a difference map and refined isotropically. C-bound H atoms were positioned geometrically (C–H = 0.93–0.96 Å) and allowed to ride on their parent atoms, with U_iso_(H) = 1.5U_eq_(C) for methyl H and 1.2U_eq_(C) for other H atoms.

### Crystal data

**Table:**

C_19_H_19_NO_2_	*F*(000) = 624
*M**_r_* = 293.35	*D*_x_ = 1.268 Mg m^−^^3^
Monoclinic, *P*2_1_/*c*	Mo *K*α radiation, λ = 0.71073 Å
Hall symbol: -P 2ybc	Cell parameters from 1546 reflections
*a* = 8.290 (5) Å	θ = 1.9–28.3°
*b* = 8.693 (5) Å	µ = 0.08 mm^−^^1^
*c* = 21.326 (5) Å	*T* = 293 K
β = 90.742 (5)°	Block, colorless
*V* = 1536.7 (13) Å^3^	0.20 × 0.17 × 0.16 mm
*Z* = 4	

### Data collection

**Table:**

Bruker SMART APEXII area-detector diffractometer	3803 independent reflections
Radiation source: fine-focus sealed tube	3050 reflections with *I* > 2σ(*I*)
graphite	*R*_int_ = 0.026
ω and φ scans	θ_max_ = 28.3°, θ_min_ = 1.9°
Absorption correction: multi-scan (*SADABS*; Bruker, 2008)	*h* = −9→11
*T*_min_ = 0.984, *T*_max_ = 0.987	*k* = −9→11
14325 measured reflections	*l* = −28→28

### Refinement

**Table:**

Refinement on *F*^2^	Primary atom site location: structure-invariant direct methods
Least-squares matrix: full	Secondary atom site location: difference Fourier map
*R*[*F*^2^ > 2σ(*F*^2^)] = 0.046	Hydrogen site location: inferred from neighbouring sites
*wR*(*F*^2^) = 0.137	H atoms treated by a mixture of independent and constrained refinement
*S* = 1.05	*w* = 1/[σ^2^(*F*_o_^2^) + (0.0713*P*)^2^ + 0.2629*P*] where *P* = (*F*_o_^2^ + 2*F*_c_^2^)/3
3803 reflections	(Δ/σ)_max_ = 0.004
207 parameters	Δρ_max_ = 0.24 e Å^−^^3^
0 restraints	Δρ_min_ = −0.22 e Å^−^^3^

### Special details

**Table:**

Geometry. All esds (except the esd in the dihedral angle between two l.s. planes) are estimated using the full covariance matrix. The cell esds are taken into account individually in the estimation of esds in distances, angles and torsion angles; correlations between esds in cell parameters are only used when they are defined by crystal symmetry. An approximate (isotropic) treatment of cell esds is used for estimating esds involving l.s. planes.
Refinement. Refinement of *F*^2^ against ALL reflections. The weighted *R*-factor wR and goodness of fit *S* are based on *F*^2^, conventional *R*-factors *R* are based on *F*, with *F* set to zero for negative *F*^2^. The threshold expression of *F*^2^ > σ(*F*^2^) is used only for calculating *R*-factors(gt) etc. and is not relevant to the choice of reflections for refinement. *R*-factors based on *F*^2^ are statistically about twice as large as those based on *F*, and *R*- factors based on ALL data will be even larger.

### Fractional atomic coordinates and isotropic or equivalent isotropic displacement parameters (Å^2^)

**Table:**

	*x*	*y*	*z*	*U*_iso_*/*U*_eq_	
O1	1.12605 (11)	0.25969 (10)	0.57699 (4)	0.0444 (2)	
O2	0.32475 (14)	0.26609 (14)	0.86787 (5)	0.0676 (3)	
N1	0.93165 (14)	0.40700 (14)	0.78439 (5)	0.0436 (3)	
C2	0.78388 (15)	0.37981 (14)	0.81195 (6)	0.0396 (3)	
C3	0.72718 (17)	0.42649 (16)	0.87003 (6)	0.0468 (3)	
H3	0.7911	0.4851	0.8971	0.056*	
C4	0.57398 (18)	0.38336 (16)	0.88612 (6)	0.0487 (3)	
H4	0.5338	0.4134	0.9248	0.058*	
C5	0.47677 (16)	0.29541 (15)	0.84589 (6)	0.0451 (3)	
C6	0.53268 (16)	0.24753 (14)	0.78823 (6)	0.0407 (3)	
H6	0.4684	0.1883	0.7616	0.049*	
C7	0.68785 (15)	0.29046 (14)	0.77121 (5)	0.0368 (3)	
C8	0.78297 (14)	0.26342 (13)	0.71591 (5)	0.0360 (3)	
C9	0.75455 (14)	0.18608 (15)	0.65939 (6)	0.0381 (3)	
H9	0.6579	0.1337	0.6532	0.046*	
C10	0.86873 (15)	0.18656 (14)	0.61251 (5)	0.0381 (3)	
C11	1.01461 (15)	0.26595 (13)	0.62376 (6)	0.0364 (3)	
C12	1.25668 (16)	0.37274 (16)	0.57659 (7)	0.0472 (3)	
C13	1.31224 (18)	0.40802 (18)	0.64208 (8)	0.0561 (4)	
H13	1.4190	0.4368	0.6492	0.067*	
C14	1.21332 (16)	0.39936 (17)	0.69017 (7)	0.0493 (3)	
H14	1.2471	0.4319	0.7298	0.059*	
C15	1.05153 (14)	0.33853 (14)	0.68064 (6)	0.0377 (3)	
C16	0.93148 (14)	0.33859 (14)	0.72584 (5)	0.0369 (3)	
C17	0.23594 (19)	0.1452 (2)	0.84007 (8)	0.0625 (4)	
H17A	0.2161	0.1681	0.7966	0.094*	
H17B	0.1350	0.1338	0.8611	0.094*	
H17C	0.2963	0.0512	0.8435	0.094*	
C18	0.83971 (18)	0.10395 (18)	0.55133 (6)	0.0512 (3)	
H18A	0.8971	0.0081	0.5517	0.077*	
H18B	0.8771	0.1665	0.5174	0.077*	
H18C	0.7264	0.0846	0.5459	0.077*	
C19	1.38727 (19)	0.2969 (2)	0.53819 (8)	0.0624 (4)	
H19A	1.4205	0.2029	0.5582	0.094*	
H19B	1.4780	0.3650	0.5352	0.094*	
H19C	1.3459	0.2747	0.4969	0.094*	
C20	1.1956 (2)	0.5186 (2)	0.54519 (9)	0.0694 (5)	
H20A	1.1633	0.4962	0.5028	0.104*	
H20B	1.2800	0.5943	0.5452	0.104*	
H20C	1.1049	0.5577	0.5678	0.104*	
H1	1.005 (2)	0.469 (2)	0.7994 (8)	0.063 (5)*	

### Atomic displacement parameters (Å^2^)

**Table:**

	*U*^11^	*U*^22^	*U*^33^	*U*^12^	*U*^13^	*U*^23^
O1	0.0402 (5)	0.0453 (5)	0.0480 (5)	−0.0053 (4)	0.0115 (4)	−0.0031 (4)
O2	0.0550 (7)	0.0727 (8)	0.0759 (7)	−0.0199 (5)	0.0296 (6)	−0.0240 (6)
N1	0.0379 (6)	0.0484 (6)	0.0446 (6)	−0.0083 (5)	0.0010 (4)	−0.0095 (5)
C2	0.0392 (7)	0.0376 (6)	0.0421 (6)	−0.0015 (5)	0.0011 (5)	−0.0026 (5)
C3	0.0519 (8)	0.0456 (7)	0.0431 (6)	−0.0064 (6)	0.0028 (5)	−0.0091 (5)
C4	0.0566 (8)	0.0456 (7)	0.0443 (6)	−0.0036 (6)	0.0118 (6)	−0.0081 (5)
C5	0.0427 (7)	0.0412 (7)	0.0516 (7)	−0.0026 (5)	0.0115 (6)	−0.0022 (5)
C6	0.0379 (7)	0.0389 (6)	0.0454 (6)	−0.0027 (5)	0.0032 (5)	−0.0035 (5)
C7	0.0363 (6)	0.0348 (6)	0.0392 (6)	0.0008 (5)	0.0012 (5)	−0.0017 (5)
C8	0.0325 (6)	0.0352 (6)	0.0402 (6)	−0.0010 (4)	0.0010 (4)	0.0000 (4)
C9	0.0320 (6)	0.0406 (6)	0.0419 (6)	−0.0044 (5)	0.0003 (5)	−0.0033 (5)
C10	0.0371 (6)	0.0372 (6)	0.0400 (6)	−0.0011 (5)	0.0007 (5)	−0.0031 (5)
C11	0.0335 (6)	0.0343 (6)	0.0416 (6)	0.0012 (4)	0.0049 (5)	0.0017 (5)
C12	0.0375 (7)	0.0454 (7)	0.0589 (8)	−0.0043 (5)	0.0118 (6)	0.0042 (6)
C13	0.0379 (7)	0.0620 (9)	0.0686 (9)	−0.0139 (6)	0.0062 (6)	−0.0086 (7)
C14	0.0383 (7)	0.0537 (8)	0.0558 (7)	−0.0093 (6)	0.0009 (6)	−0.0077 (6)
C15	0.0331 (6)	0.0359 (6)	0.0443 (6)	−0.0015 (5)	0.0004 (5)	−0.0009 (5)
C16	0.0343 (6)	0.0356 (6)	0.0409 (6)	−0.0012 (4)	−0.0012 (5)	−0.0016 (5)
C17	0.0472 (9)	0.0626 (10)	0.0781 (11)	−0.0123 (7)	0.0114 (7)	−0.0048 (8)
C18	0.0496 (8)	0.0590 (9)	0.0450 (7)	−0.0091 (6)	0.0049 (6)	−0.0127 (6)
C19	0.0458 (8)	0.0706 (10)	0.0714 (10)	0.0002 (7)	0.0205 (7)	0.0012 (8)
C20	0.0619 (10)	0.0536 (9)	0.0931 (12)	−0.0001 (8)	0.0121 (9)	0.0182 (9)

### Geometric parameters (Å, °)

**Table:**

O1—C11	1.3693 (15)	C10—C18	1.5059 (17)
O1—C12	1.4625 (17)	C11—C15	1.3977 (17)
O2—C5	1.3741 (18)	C12—C13	1.497 (2)
O2—C17	1.410 (2)	C12—C19	1.517 (2)
N1—C16	1.3830 (16)	C12—C20	1.518 (2)
N1—C2	1.3859 (18)	C13—C14	1.323 (2)
N1—H1	0.872 (18)	C13—H13	0.9300
C2—C3	1.3911 (18)	C14—C15	1.4535 (19)
C2—C7	1.4050 (17)	C14—H14	0.9300
C3—C4	1.372 (2)	C15—C16	1.3945 (18)
C3—H3	0.9300	C17—H17A	0.9600
C4—C5	1.397 (2)	C17—H17B	0.9600
C4—H4	0.9300	C17—H17C	0.9600
C5—C6	1.3838 (18)	C18—H18A	0.9600
C6—C7	1.3920 (19)	C18—H18B	0.9600
C6—H6	0.9300	C18—H18C	0.9600
C7—C8	1.4463 (17)	C19—H19A	0.9600
C8—C9	1.3975 (16)	C19—H19B	0.9600
C8—C16	1.4075 (18)	C19—H19C	0.9600
C9—C10	1.3856 (17)	C20—H20A	0.9600
C9—H9	0.9300	C20—H20B	0.9600
C10—C11	1.4102 (18)	C20—H20C	0.9600
			
C11—O1—C12	118.95 (10)	C13—C12—C20	109.71 (14)
C5—O2—C17	118.11 (12)	C19—C12—C20	111.18 (13)
C16—N1—C2	108.60 (10)	C14—C13—C12	121.68 (13)
C16—N1—H1	126.3 (11)	C14—C13—H13	119.2
C2—N1—H1	124.4 (11)	C12—C13—H13	119.2
N1—C2—C3	129.69 (12)	C13—C14—C15	119.48 (13)
N1—C2—C7	109.20 (11)	C13—C14—H14	120.3
C3—C2—C7	121.11 (12)	C15—C14—H14	120.3
C4—C3—C2	117.87 (12)	C16—C15—C11	116.73 (11)
C4—C3—H3	121.1	C16—C15—C14	124.66 (12)
C2—C3—H3	121.1	C11—C15—C14	118.49 (11)
C3—C4—C5	121.62 (12)	N1—C16—C15	129.19 (11)
C3—C4—H4	119.2	N1—C16—C8	109.03 (11)
C5—C4—H4	119.2	C15—C16—C8	121.78 (11)
O2—C5—C6	124.51 (13)	O2—C17—H17A	109.5
O2—C5—C4	114.60 (12)	O2—C17—H17B	109.5
C6—C5—C4	120.87 (13)	H17A—C17—H17B	109.5
C5—C6—C7	118.19 (12)	O2—C17—H17C	109.5
C5—C6—H6	120.9	H17A—C17—H17C	109.5
C7—C6—H6	120.9	H17B—C17—H17C	109.5
C6—C7—C2	120.34 (12)	C10—C18—H18A	109.5
C6—C7—C8	133.19 (11)	C10—C18—H18B	109.5
C2—C7—C8	106.47 (11)	H18A—C18—H18B	109.5
C9—C8—C16	119.37 (11)	C10—C18—H18C	109.5
C9—C8—C7	133.94 (11)	H18A—C18—H18C	109.5
C16—C8—C7	106.68 (10)	H18B—C18—H18C	109.5
C10—C9—C8	120.78 (11)	C12—C19—H19A	109.5
C10—C9—H9	119.6	C12—C19—H19B	109.5
C8—C9—H9	119.6	H19A—C19—H19B	109.5
C9—C10—C11	118.11 (11)	C12—C19—H19C	109.5
C9—C10—C18	121.38 (11)	H19A—C19—H19C	109.5
C11—C10—C18	120.51 (11)	H19B—C19—H19C	109.5
O1—C11—C15	120.52 (11)	C12—C20—H20A	109.5
O1—C11—C10	116.22 (11)	C12—C20—H20B	109.5
C15—C11—C10	123.10 (11)	H20A—C20—H20B	109.5
O1—C12—C13	110.56 (11)	C12—C20—H20C	109.5
O1—C12—C19	104.16 (12)	H20A—C20—H20C	109.5
C13—C12—C19	112.32 (13)	H20B—C20—H20C	109.5
O1—C12—C20	108.74 (12)		
			
C16—N1—C2—C3	−179.35 (13)	C9—C10—C11—O1	178.11 (11)
C16—N1—C2—C7	1.02 (15)	C18—C10—C11—O1	−1.39 (17)
N1—C2—C3—C4	179.97 (14)	C9—C10—C11—C15	2.60 (19)
C7—C2—C3—C4	−0.4 (2)	C18—C10—C11—C15	−176.90 (12)
C2—C3—C4—C5	0.0 (2)	C11—O1—C12—C13	36.86 (16)
C17—O2—C5—C6	19.3 (2)	C11—O1—C12—C19	157.72 (12)
C17—O2—C5—C4	−162.32 (14)	C11—O1—C12—C20	−83.65 (15)
C3—C4—C5—O2	−177.91 (13)	O1—C12—C13—C14	−29.5 (2)
C3—C4—C5—C6	0.5 (2)	C19—C12—C13—C14	−145.39 (16)
O2—C5—C6—C7	177.72 (13)	C20—C12—C13—C14	90.42 (18)
C4—C5—C6—C7	−0.5 (2)	C12—C13—C14—C15	6.7 (2)
C5—C6—C7—C2	0.09 (19)	O1—C11—C15—C16	−179.54 (10)
C5—C6—C7—C8	−179.29 (13)	C10—C11—C15—C16	−4.22 (18)
N1—C2—C7—C6	−179.94 (11)	O1—C11—C15—C14	−3.37 (18)
C3—C2—C7—C6	0.4 (2)	C10—C11—C15—C14	171.96 (12)
N1—C2—C7—C8	−0.40 (14)	C13—C14—C15—C16	−172.97 (14)
C3—C2—C7—C8	179.92 (12)	C13—C14—C15—C11	11.2 (2)
C6—C7—C8—C9	0.6 (2)	C2—N1—C16—C15	178.26 (13)
C2—C7—C8—C9	−178.84 (13)	C2—N1—C16—C8	−1.24 (14)
C6—C7—C8—C16	179.10 (13)	C11—C15—C16—N1	−176.68 (12)
C2—C7—C8—C16	−0.34 (13)	C14—C15—C16—N1	7.4 (2)
C16—C8—C9—C10	−1.93 (18)	C11—C15—C16—C8	2.77 (18)
C7—C8—C9—C10	176.42 (12)	C14—C15—C16—C8	−173.14 (12)
C8—C9—C10—C11	0.59 (18)	C9—C8—C16—N1	179.73 (11)
C8—C9—C10—C18	−179.91 (12)	C7—C8—C16—N1	0.97 (14)
C12—O1—C11—C15	−22.17 (17)	C9—C8—C16—C15	0.18 (18)
C12—O1—C11—C10	162.20 (11)	C7—C8—C16—C15	−178.58 (11)

### Hydrogen-bond geometry (Å, °)

**Table:**

Cg1 is the centroid of the N1/C2/C7/C8/C16 ring and Cg4 is the centroid of the C8–C11/C15/C16 ring.

**Table:**

*D*—H···*A*	*D*—H	H···*A*	*D*···*A*	*D*—H···*A*
C3—H3···O1^i^	0.93	2.54	3.333 (2)	143
N1—H1···Cg4^ii^	0.872 (18)	2.744 (17)	3.528 (2)	149.7 (14)
C17—H17C···Cg1^iii^	0.96	3.08	3.489 (3)	107
C17—H17C···Cg4^iii^	0.96	3.00	3.514 (3)	115

Symmetry codes: (i) −*x*+2, *y*+1/2, −*z*+3/2; (ii) −*x*+2, *y*−1/2, −*z*+1/2; (iii) −*x*+1, *y*+1/2, −*z*+1/2.
